# Impact of endometrial compaction on reproductive outcomes after cryotransfer of euploid embryos in a modified natural cycle: protocol for a prospective cohort study

**DOI:** 10.3389/fendo.2023.1285040

**Published:** 2023-11-09

**Authors:** Esperanza De la Torre Perez, Maria Concepción Carratalá-Munuera, Juan Carlos Castillo-Farfán, Belén Lledó-Bosch, Belén Moliner-Renau, Andrea Bernabeu-García, Rafael Bernabeu-Pérez

**Affiliations:** ^1^ Medical Department, Bernabeu Institute, Madrid, Spain; ^2^ Clinical Medicine Department, School of Medicine. University Miguel Hernández de Elche, Alicante, Spain; ^3^ Medical Department, Bernabeu Institute, Alicante, Spain; ^4^ University Chair of Community Medicine and Reproductive Health, Miguel Hernandez University of Elche, Alicante, Spain

**Keywords:** assisted reproduction technology, ectopic pregnancy, endometrial compaction, *in vitro* fertilization-embryo transfer, endometrial thickness, IVF, placental complications

## Abstract

**Introduction:**

Embryo implantation is a complex and poorly understood process. Most studies to date have focused on the analysis of the endometrium at the end of the estrogenic phase, while the available data on its importance after secretory transformation are limited and inconsistent. Current evidence does not allow for a conclusive interpretation of the changes observed in the pre-implantation endometrium, whether in the natural or replacement cycle, and their relevance in the development of a pregnancy or the implications for clinical practice.

**Methods:**

Multicenter prospective observational cohort study. Based on our sample size calculation, the study group will consist of 206 women (exposed or “compaction” group: 103 women with a decrease of ≥ 5% in endometrial thickness between the estrogenic phase and the day of embryo transfer; non-exposed “non-compaction” group: 103 women with similar or greater endometrial thickness between these time points). The main objective of this study is to compare the ongoing pregnancy rates in natural cycles for euploid embryo transfer in patients who present endometrial compaction at the time of transfer versus those who with a stable or greater endometrial thickness with respect to the estrogenic phase. The estimated duration of the study is 30 months. Inclusion criteria are: 18 to 50 years of age, with primary or secondary infertility, subjected to endometrial preparation in a modified natural cycle for transfer of a genetically euploid blastocyst, from their own oocyte or oocyte donation, with a normal uterine cavity. Exclusion criteria are: uterine or endometrial disease (e.g., multiple myomatosis, severe adenomyosis, Asherman syndrome, refractory endometrium), conditions that prevent correct ultrasound assessment (tilted uterus), or a history of recurrent implantation failure or repeated miscarriages.

**Discussion:**

The findings from this study will provide valuable insights into the potential influence of the “endometrial compaction” phenomenon on reproductive outcomes during natural cycle endometrial preparation. By examining this aspect, we aim to contribute to a better understanding of the factors that may impact successful outcomes in fertility treatments.

## 1 Introduction

Embryo implantation is a complex and poorly understood process, in which critical cross-talk must be established between the developing embryo and the receptive endometrial surface (1). Various hypotheses have been put forward about the conditions necessary for a receptive endometrium, among which is endometrial thickness. In that line, ultrasound monitoring of the endometrial cycle is currently the most widely used method to pinpoint the ideal moment for embryo transfer in the so-called “implantation window” (2).

Most studies to date have analyzed the endometrium at the end of the estrogenic phase, accepting that trilaminar morphology with a thickness of 7 mm to 12 mm is associated with a higher pregnancy rate (3–9). In contrast, an endometrial thickness under 7 mm compromises the prognosis of the transfer (3–5) and reduces the odds of implantation, clinical pregnancy, and a live birth (6, 10), along with increasing the risk of adverse obstetric outcomes derived from deficient placentation (11–19). However, the available data on the importance of the endometrium after secretory transformation are limited and inconsistent (20, 21).

Approximately half of women present reduced endometrial thickness around embryonic implantation relative to that measured in the estrogenic phase, a phenomenon known as endometrial compaction. In recent years, several studies have tried to determine if this event is a factor in reproductive outcomes (22–32). Some authors have found no differences associated with endometrial compaction in Frozen embryo transfer (FET) during a replacement cycle with estro-progesterone therapy (22–24), whereas others have observed a positive association between compaction and pregnancy rates (25–27). Regarding the natural cycle, research interest has been increasing significantly, but so far, the information is even more limited and discordant. One retrospective study from 2015 to 2019 (28) described compaction as more frequent in natural cycles than in replacement ones, associating it with a negative impact on the pregnancy rate. However, another study (29) found more compaction in the replacement cycle, but no significant association with the pregnancy rate. Subsequently, a prospective investigation (30) analyzed euploid embryo transfers in replacement, stimulated, and natural cycles, finding no differences in endometrial compaction. Two studies have recently been carried out during natural cycles (31, 32). In one (31), endometrial expansion was associated with a slight increase in clinical pregnancy, which was not reflected in changes in live births. However, a later study (32) reported that endometrial compaction was associated with a better pregnancy rate.

One reason for the incomplete transformation of the endometrium in the secretory phase may originate in an alteration in the estradiol-progesterone ratio, which occurs in certain situations such as ovarian hyperstimulation. One study (33) found that compaction was inversely proportional to the response to ovarian stimulation, although authors did not find a clear association between these changes and the pregnancy rate.

Furthermore, it is unclear whether the endometrial changes that occur before implantation are relevant to gestational complications. To date, only one study has identified compaction as a protective factor for ectopic pregnancy (34). However, a subsequent study (35) found no association between endometrial compaction prior to embryo transfer and preterm birth or placenta-mediated pregnancy complications. Current evidence does not allow for a conclusive interpretation of the changes observed in the pre-implantation endometrium, whether in the natural or replacement cycle, and their relevance in the development of a pregnancy or the implications for clinical practice. Therefore, the main objective of this study is to compare reproductive outcomes (ongoing pregnancy rate) in a homogeneous sample of patients who undergo euploid embryo transfer in a modified natural cycle, according to whether they present endometrial compaction at the time of transfer or show a stable or greater endometrial thickness relative to the estrogenic phase. Likewise, we will analyze whether endometrial compaction is associated with serum progesterone levels on the day of the transfer, and we will assess the variation in serum progesterone on the day of the pregnancy test and its impact on reproductive outcomes: biochemical pregnancy, clinical pregnancy, ongoing pregnancy, and early pregnancy loss rates.

## 2 Methods

### 2.1 Design

**Figure 1:**
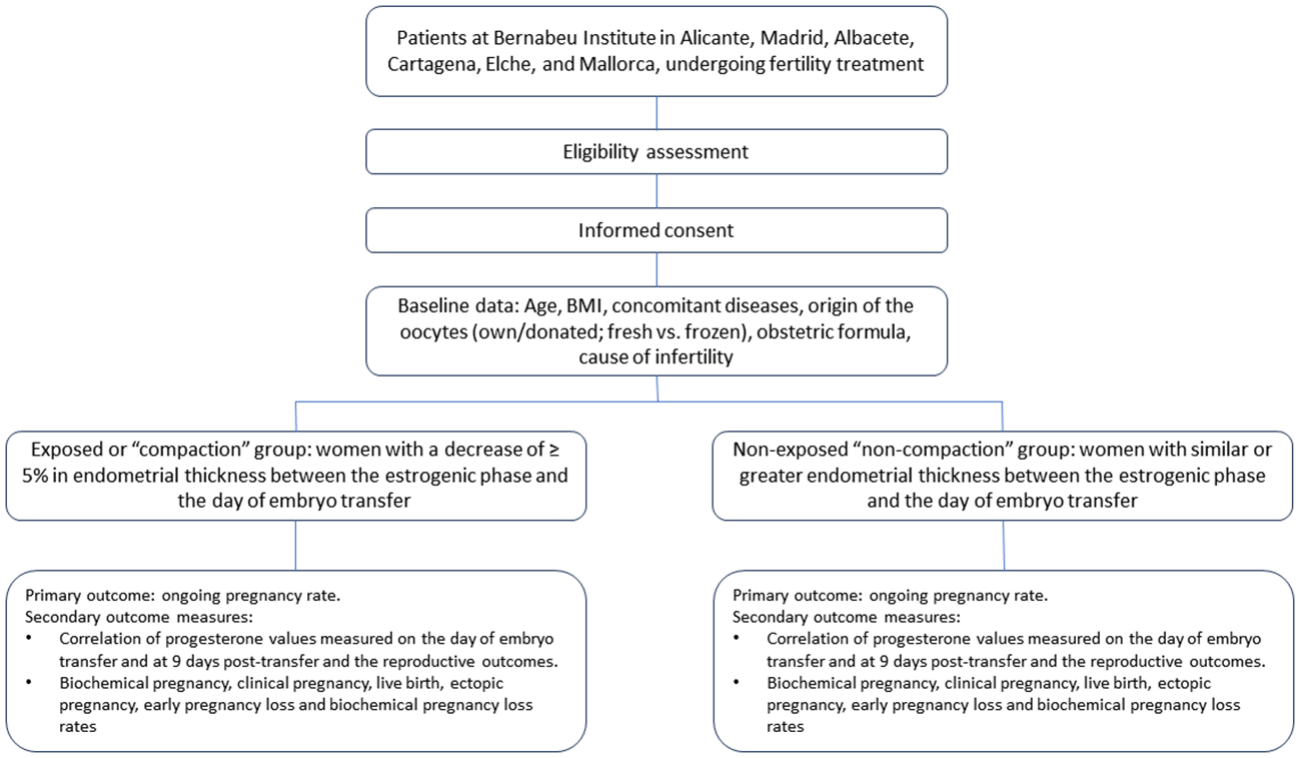
Flowchart of the study design.

This is a multicenter, prospective observational cohort study, which will be performed at the different centers of the Bernabeu Institute in Spain, specifically in Alicante, Madrid, Albacete, Cartagena, Elche, and Mallorca. The ethics committee of the Alicante General University Hospital approved the study (committee code 22/053Tut, **Supplementary Material 1**). A flowchart of this study design can be seen in **Figure 1**.

### 2.2 Study population

The sample will be drawn from patients at the Bernabeu Institute in Alicante, Madrid, Albacete, Cartagena, Elche, and Mallorca, undergoing fertility treatment that includes endometrial preparation in a modified natural cycle for transfer of previously analyzed frozen and euploid embryos, and who meet the inclusion criteria: aged 18 to 50 years, with primary or secondary infertility, with a normal uterine cavity, undergoing endometrial preparation in a modified natural cycle for single embryo transfer in the blastocyst state from own oocyte or oocyte donation cycles, who had normal results on preimplantation genetic testing for aneuploidy (PGT-A) via trophectoderm biopsy. Exclusion criteria were: uterine or endometrial disease (multiple myomatosis [>3 fibroids of > 3 cm], adenomyosis, Asherman syndrome); difficulties in correctly measuring endometrial thickness due to a retroverted or tilted uterus; a history of recurrent implantation failure (3 or more transferred blastocysts of good quality, from their own oocyte [<35 years] or oocyte donation); recurrent early Pregnancy Loss (the loss of two or more pregnancies before 10 weeks of gestational age (36) and suboptimal endometrial response (endometrium < 6 mm on the day of ovulation triggering). (**Table 1**)

**Table 1:** Inclusion/exclusion criteria.

Inclusion criteria
Age 18 to 50 years
Primary or secondary infertility
Normal uterine cavity
Endometrial preparation in a modified natural cycle
Undergoing single embryo transfer in the blastocyst state from their own oocyte or oocyte donation
Normal results on preimplantation genetic testing for aneuploidy (PGT-A) via trophectoderm biopsy
Exclusion criteria
Uterine or endometrial disease (myomatosis [>3 fibroids of > 3 cm], adenomyosis, Asherman syndrome)
Difficulties in correctly measuring endometrial thickness (e.g. due to a retroverted or tilted uterus)
History of recurrent implantation failure (3 or more transferred blastocysts of good quality)
History of recurrent early pregnancy loss
Suboptimal endometrial response (endometrium < 6 mm on the day of ovulation triggering)

Eligible patients who sign informed consent will be divided into two cohorts: the exposed (or compaction) group and the non-exposed (non-compaction) group, depending on the endometrial thickness on the day of embryo transfer, as measured by transvaginal ultrasound:

- The compaction group will comprise patients who present a decrease of 5% or more in endometrial thickness on the day of embryo transfer with respect to the estrogenic phase.
- The non-compaction group will be made up of women presenting similar or greater endometrial thickness on the day of the transfer with respect to the estrogenic phase, measured with transvaginal ultrasound.

We defined compaction percentage to avoid minor measurement variations and according to previous studies using similar values (24, 25, 28, 32).

### 2.3 Sampling

Researchers at the assisted reproduction services of the Bernabeu Institute centers will be responsible for recruitment during their clinical practice, through opportunistic sampling of the patients undergoing frozen embryo transfer after PGT-A. Together with the embryo transfer consent, patients will receive information on the purpose of the study and be asked to sign informed consent as a condition for participating (**Supplementary Material 2**). The endometrial preparation treatment will not differ from usual practice.

### 2.4 Variables

Data collection will commence after both the patient and the researcher have signed informed consent. A purpose-designed data collection notebook will be designed for the study. The variables under study will be incorporated into an anonymized and encrypted database for subsequent statistical analysis. The main explanatory (exposure) variable is endometrial compaction, defined as a reduction in the thickness of the endometrium of 5% or more from the day of ovulation induction to the day of embryo transfer. Other variables include:

#### 2.4.1 Baseline patient parameters from the electronic medical record

- Age at the time of transfer.
- Standardized body mass index.
- Concomitant diseases (hypertension, hypothyroidism, diabetes, autoimmune diseases…).
- Origin of the oocytes: own gametes/donated gametes; fresh vs. frozen.
- Obstetric formula.
- Cause of infertility:
  - Male factor: male diagnosed with seminal or urological
  - problems causing infertility.
  - Uterine factor: presence of uterine disease (fibroids, synechiae, adenomyosis) causing infertility.
  - Tubal factor: obstruction of fallopian tubes.
  - Ovarian factor: endometriosis, low ovarian reserve, previous ovarian surgery.
  - Unknown cause: not included in any of the above.
  - Mixed cause: presence of 2 or more factors.

#### 2.4.2 Prospective variables: cycle follow-up

- Follicular phase length until ovulation induction (days).
- Endometrial thickness (mm), as measured by vaginal ultrasound in the follicular phase prior to ovulation induction (recombinant hCG 6500 subcutaneous IU).
- Endometrial thickness (mm) in the secretory phase (7 days after administration of recombinant hCG), at the time of embryo transfer, as measured by vaginal ultrasound*.
- Serum progesterone on the day of embryo transfer and at 9 days post-transfer.
- Serum b-hCG at 9 days after the embryo transfer, according to standard protocol.

#### 2.4.3 Definitions for reproductive outcomes

Definitions for reproductive outcomes were (36, 37):

- Biochemical pregnancy: A pregnancy diagnosed only by the detection of beta hCG in serum 9 days after the embryo transfer.
- Clinical pregnancy: A pregnancy diagnosed by ultrasonographic visualization of one or more gestational sacs or definitive clinical signs of pregnancy. In addition to intra-uterine pregnancy, it includes a clinically documented ectopic pregnancy.
- Ongoing pregnancy: the presence of positive heartbeat as seen by sonography at 10 weeks gestational age.
- Live birth: 22 completed weeks of gestational age.
- Ectopic pregnancy: ultrasonic or surgical visualization of a pregnancy outside of the endometrial cavity.
- Early pregnancy loss: spontaneous pregnancy demise before 10 weeks of gestational age (before 8th developmental week).
- Biochemical pregnancy loss: spontaneous pregnancy demise based on decreasing serum b-hCG levels, without an ultrasound evaluation.

*Both ultrasounds will be performed by the attending gynecologist. In the cases of international patients who perform the follicular faze scan outside the center, a standardized data collection sheet (**Supplementary Material 3**) will be used, and the attending gynecologist will evaluate both this and the ultrasound images according to standard protocols.

### 2.5 Natural cycle monitoring

Women with regular menstrual cycles (28 ± 7 days) will undergo transvaginal ultrasound between days 7 to 10 of their menstrual cycle, adjusted based on cycle length. This procedure aims to monitor endometrial and follicular growth and will be repeated every two days as required. Ovulation will be induced using 6500 IU of hCGr (Ovitrelle^®^; NV Organon) when ultrasound reveals an endometrial thickness of 7 mm or more and a follicle measuring 17-20 mm, aligning with standard clinical practice (38–40). Patients will receive a daily vaginal dose of 400 mg progesterone pessaries at bed time (Cyclogest^®^; Gedeon Richter,Budapest, Hungary) starting two-days after hCG administration and continued until 7 weeks of gestation if pregnancy is achieved (41).

#### 2.5.1 Outcome measures

##### 2.5.1.1 Primary outcome measure

The primary outcome for the comparison of the two groups is the ongoing pregnancy rate.

##### 2.5.1.2 Secondary outcome measures

Correlation of progesterone values measured on the day of embryo transfer and at 9 days post-transfer and the reproductive outcomes.

Biochemical pregnancy, clinical pregnancy, live birth, ectopic pregnancy, early pregnancy loss and biochemical pregnancy loss rates.

#### 2.5.2 Sample size calculation and statistical analysis

With an expected proportion of live births in the non-compaction group of 50% and in the compaction group of 70%, and taking into account a two-sided significance level of 0.05 and a power of 80%, the number of women required in each group would be 93. Assuming an attrition rate of 15%, 103 women are needed in each group, for a total sample size of 206.

In the descriptive analysis, qualitative variables will be expressed as frequency and percentage, and quantitative variables as measures of central tendency and dispersion. For the univariable analysis, the Chi-square test or Fisher’s exact test will be used to compare qualitative variables. The normality of the quantitative variables will be checked using the Kolmogorov-Smirnov test, and in the case of a non-parametric distribution, a log transformation will be performed. If the distribution is normal, the student’s t test will be used for comparison. P values of less than 0.05 will be considered statistically significant. Variables that do not meet the criterion of normality will be analyzed using the Wilcoxon-Mann-Whitney test. Multivariable analyses will be carried out using linear or binary logistic regression to control for potential confounders. Cases will be entered into a database and analyzed using the statistical package SPSS version 20.0 for Windows (SPSS Inc. Chicago. IL).

## 3 Discussion

Currently, there is no solid evidence that allows us to interpret whether the changes observed in the pre-implantation endometrium influence pregnancy outcomes. Although scientific interest in this area has increased dramatically in recent years, the results published to date are highly heterogeneous, probably due to differences in the study population; endometrial preparation protocols; type of ultrasounds used for the assessment of the endometrium (abdominal vs. vaginal); and number, quality and stage of the transferred embryos; among other differences. Nevertheless, it is plausible that the pre-implantation endometrium may play a role in the subsequent development of gestational complications related to placentation. To date, only two retrospective studies have looked into this question: the first (34) identified compaction as a protective factor for ectopic pregnancy, while the second (35) found no association between this event and preterm birth or placenta-mediated pregnancy complications. The present study would be the first to provide prospective evidence on the possible impact of endometrial compaction on obstetric complications such as early miscarriage, biochemical pregnancy loss, and ectopic pregnancy, including detailed baseline and clinical data from the patients. In addition, an exploratory study of complications in advanced pregnancy and childbirth could be considered.

One potential limitation of this study resides in the fact that different professionals will perform the ultrasound scans, and in the case of international patients, these will be professionals outside the center, which could introduce a measurement bias. However, measures will be taken to minimize this bias by requesting imaging results from patients whose ultrasound is performed outside the center, as done in routine practice. Images of poor quality that cannot be evaluated will be excluded from the study.

Regarding the strengths, this study will be the first prospective analysis of reproductive outcomes from euploid embryos in natural cycles, comparing patients who present endometrial compaction versus stable or increased endometrial thickness at the time of transfer. In addition, we will assess the association between endometrial compaction and serum progesterone levels, variations in serum progesterone and its impact on pregnancy outcomes, implantation rate, clinical pregnancy, clinical abortion, and biochemical abortion. The prospective design will allow a careful selection of the sample and rigorous collection of patient variables. In addition, its multicenter nature will favor the generalizability of the results. Furthermore, the sample will include national and international patients, and all necessary resources are available, with no need for modifying usual clinical practice.

The primary purpose of this study is to assess reproductive outcomes, specifically the ongoing pregnancy rate, in a homogeneous sample of patients undergoing euploid embryo transfer using a modified natural cycle. A prospective analysis will be performed to investigate whether the observed changes in endometrial development and serum progesterone levels in the natural cycle are relevant to the development of a pregnancy. This approach will contribute to improving our understanding of the ideal circumstances for embryo implantation and its application in clinical practice. In addition, the correlation between these factors and serum progesterone levels will be discussed, which could provide an additional avenue for outcome evaluation. The results obtained from this clinical research will be reviewed and discussed by the research team for subsequent publication and dissemination.

## Ethics statement

The ethics committee of the Alicante General University Hospital (22/053Tut) approved this study. The results obtained as a result of clinical research will be reviewed and discussed by the research team for subsequent publication and dissemination. The study will be carried out in strict compliance with international research ethics norms. Before including any study participant, the ethics committee of Alicante General University Hospital approved the protocol, the information sheet that will be given to the participants, and the informed consent form that will be used.

## Author contributions

EP: Conceptualization, Methodology, Supervision, Writing – original draft, Writing – review and editing. MC-M: Conceptualization, Methodology, Supervision, Writing – original draft, Writing – review and editing. JC-F: Conceptualization, Methodology, Supervision, Writing – original draft, Writing – review and editing. BL-B: Investigation, Writing – original draft, Writing – review and editing. MB-R: Conceptualization, Methodology, Supervision, Writing – original draft, Writing – review and editing. A-G: Conceptualization, Methodology, Supervision, Writing – original draft, Writing – review and editing. RBP: Conceptualization, Methodology, Supervision, Writing – original draft, Writing – review and editing.
