# Supplementary material for: Impact of endometrial compaction on reproductive outcomes after cryotransfer of euploid embryos in a modified natural cycle: protocol for a prospective cohort study
Source: Front Endocrinol (Lausanne). 2023 Nov 9;14:1285040. doi: 10.3389/fendo.2023.1285040 (PMC10666753; doi:10.3389/fendo.2023.1285040)
Supplement: Supplementary file 1 [file DataSheet_1.pdf]

frontiers

Supplementary Material

Supplementary material 1- Approval from research ethics committee at the Alicante General University Hospital

GENERALITAT VALENCIANA

Hospital Universitari de Sant Joan d'Alacant

COMITÉ DE ÉTICA DE LA INVESTIGACIÓN DEL HOSPITAL UNIVERSITARIO SAN JUAN DE ALICANTE

DRA. ROSARIO MARTÍN GONZÁLEZ, Presidenta del Comité de Ética de la Investigación del Hospital Universitario San Juan de Alicante,

CERTIFICA

Que este Comité, en su reunión de fecha 26 de Julio de 2022, ha evaluado la propuesta de la investigadora **Dra. Esperanza de la Torre Pérez,** del Instituto Bernabeu de Alicante, para que sea realizado el proyecto de investigación titulado **"RELEVANCIA DE LA COMPACTACIÓN ENDOMETRIAL EN CICLO NATURAL PARA TRANSFERENCIA DE EMBRIÓN EUPLOIDE."** Código de Comité: **22/053 Tut.**

y que considera que:

- Se cumplen los requisitos necesarios de idoneidad del protocolo en relación con los objetivos del estudio.
- La capacidad del investigador y los medios disponibles son apropiados para llevar a cabo el estudio.
- Son adecuados los procedimientos para obtener el consentimiento informado.
- El tratamiento de la información del estudio se realizará conforme a la legislación vigente de protección y confidencialidad de los datos en relación a los métodos, riesgos y tratamiento de los mismos tal y como se contempla en la Ley Orgánica 3/2018, de 5 de diciembre, de Protección de Datos Personales y garantía de los derechos digitales, el Reglamento 2016/679 del Parlamento Europeo y del Consejo, de 27 de abril de 2016.

y que este Comité da su aprobación a dicho estudio para que sea realizado por la **Dra. Esperanza de la Torre Pérez,** del Instituto Bernabeu de Alicante.

Lo que firmo en San Juan, a 29 de Julio de 2022

PRESIDENTA DEL CEI

Fdo.: Dra. Rosario Martín González

Supplementary Material 2- Information sheet and informed consent form provided to patient

INFORMED CONSENT FOR PARTICIPATION IN THE CLINICAL STUDY ENTITLED:

Impact of endometrial compaction on reproductive outcomes after cryotransfer of euploid embryos in a natural cycle

1.1 Information sheet

We would like to invite your voluntary participation in the clinical study entitled "Impact of endometrial compaction on reproductive outcomes after cryotransfer of euploid embryos in a natural cycle."

With this study we intend to deepen our knowledge of the changes that occur in the postovulatory endometrium in patients undergoing embryo transfer in the natural cycle, investigating whether there is an association between changes in endometrial thickness after ovulation and the positive pregnancy rate.

This is not an experimental study, which means it does NOT imply any change in your medical treatment nor does it entail any risk for you. Rather, aspects of your case will simply be analyzed, and the only additional intervention would be a transvaginal ultrasound to examine the endometrium before the transfer.

If the treatment results in pregnancy, after the delivery it is possible that we will contact you by telephone for information about the evolution of the pregnancy.

Your contribution is completely anonymous, confidential, and voluntary, and it can be revoked at any time. This study complies with Organic Law 3/2018 of 5 December on the Protection of personal data and guarantee of digital rights, and with all pertinent ethical norms, having been approved by the research ethics committee of the Alicante General University Hospital.

The sole promoter and funder of this study is Bernabéu Institute, and it will be carried out exclusively at our centers, with Dr. Esperanza de la Torre serving as the principal investigator. If you have any questions or comments related to the conduct of the investigation, you can contact her:

Dra. Esperanza de la Torre Pérez.

Gynecologist specializing in assisted reproductive therapies (918 333 882)

1.2 Informed consent form

I, ……………………..................................... ...., with national identification number ..………… ………, of legal age, residing at………………………………….. grant my consent to participate in the study entitled: "Impact of endometrial compaction on reproductive outcomes after cryotransfer of euploid embryos in a natural cycle."

- I have read the document, which includes clear and precise information on the investigation, regarding:
    - Study objectives.
    - Mode of participation.
    - Voluntary nature of my participation.
    - Risks and benefits.
    - Right to know the results.
    - Right to withdraw from the study at any time without the need to give explanations or see changes in my medical care.
    - Confidentiality.
    - Information on the researcher, the sponsor, and the scientific ethics committee.

I understand the declarations contained in this document and the need to declare my consent, which I give freely and voluntarily, and understand that I will receive a copy of this document as soon as it is signed.

I have spoken with ………………………………………………………………………………….., who provided me with all the necessary information, and I have been able to ask questions.

I agree to provide data on how my pregnancy has progressed and on the health of my baby after delivery.

Participant signature **Researcher signature**

Supplementary material 3- Form for performing ultrasound in the estrogenic phase

Follow-up ultrasound

Date:

The patient…………………………………………………………..is undergoing fertility treatment at our center and requires an ultrasound to monitor her response to treatment.

Please include the following details:

Endometrial thickness ……………………………………………………………………………………….

Appearance of the endometrium (trilaminar, homogeneous, etc.)……………………………….

Ovaries: highlight in particular the presence of follicles larger than 10mm.

- Right ovary…………………………………………………………………………

- Left ovary…………………………………………………………………………

Observations:

Sonographer:
